# Development and validation of the WEll-being and Satisfaction of CAREgivers of Children with Diabetes Questionnaire (WE-CARE)

**DOI:** 10.1186/1477-7525-6-3

**Published:** 2008-01-18

**Authors:** Joseph C Cappelleri, Robert A Gerber, Teresa Quattrin, Rosemarie Deutschmann, Xuemei Luo, Robert Arbuckle, Linda Abetz

**Affiliations:** 1 Pfizer Global Research and Development, Groton, CT, USA; 2 Women's and Children's Hospital of Buffalo, Buffalo, NY, USA; 3 State University of New York, Buffalo, NY, USA; 4 Deutschmann and Company, Mt. Sheridan, Australia; 5 Mapi Values, Bollington, UK

## Abstract

**Background:**

This study was designed to develop a diabetes-specific questionnaire on parents' quality of life and satisfaction with their child's diabetes treatment, the **WE**ll-being and Satisfaction of **CARE**givers of Children with Diabetes Questionnaire, and to conduct psychometric validation of the WE-CARE.

**Methods:**

Parents of 116 children aged 6 to 11 years were enrolled in the United States. Children had type 1 diabetes mellitus for > 1 year, had been treated with subcutaneous insulin for ≥ 2 months, and had a recent glycosylated hemoglobin (HbA_1C_) measurement. Recruiting clinicians provided clinical information on the children. Over a two-week period, parents completed WE-CARE (initial 68 items) and two other questionnaires (the 36-item Short Form of the Medical Outcomes Study and the 50-item Child Health Questionnaire-Parent Form) twice.

**Results:**

A literature review and one-on-one interview with caregivers and pediatricians led to the development of a draft questionnaire consisting of 68 items. Factor analysis suggested retention of 37 of the 68 initial items grouped into four multi-item scales (Psychosocial Well-being, Ease of Insulin Use, Treatment Satisfaction, and Acceptance of Insulin Administration as well as a Total Score). The four multi-item domains of WE-CARE were found to be psychometrically robust – they had negligible floor and ceiling effects, excellent internal consistency and test-retest reliability, high item-discriminant validity and good concurrent, divergent, known-group and clinical validity. Moderate interscale correlations among the four WE-CARE domains indicated that the concepts they measure were related but distinct.

**Conclusion:**

These data suggest that WE-CARE provides a reliable and valid measure of parents' well-being and treatment satisfaction related to their child's diabetes. While these results show promise, additional validation of WE-CARE is warranted.

## Background

The US National Institute of Diabetes and Digestive and Kidney Diseases has suggested that one in every 400 to 600 children is affected by type 1 diabetes mellitus and its associated risk factors [1]. By adolescence, children with type 1 diabetes typically receive three or more insulin injections per day [2], placing a substantial burden on their parents. American Diabetes Association guidelines emphasize that care of this population requires integrated management of the complicated physical and emotional needs of children and adolescents as well as of their families [3].

Previous studies have shown that parents experience higher levels of stress in caring for a child with diabetes than in caring for a healthy child [3-7]. However, there has been a dearth of studies to quantitatively assess either the well-being of parents of children with type 1 diabetes or their satisfaction with their child's diabetes regimen. The paucity of information in this area is not surprising, as there have been a very limited number of instruments measuring these factors.

The few relevant instruments that exist have some limitations. For instance, the Parents Diabetes Quality of Life Questionnaire (PDQOL) is a modified version of Diabetes Quality of Life measure [8]. It assesses parents' perceptions of the effects of their child's diabetes, with three subscales that assess parental life satisfaction as affected by the child's diabetes, impact of the child's diabetes, and disease-related worries. As a measure of quality of life, the PDQOL does not assess parents' experience with diabetic treatment, and the questionnaire's psychometric validation was limited to internal consistency reliability and content validity.

The Pediatric Inventory for Parents, which has been used for mothers of children with type 1 diabetes [9,10], is a generic questionnaire that was not developed for parents of children with diabetes. It does not measure parents' satisfaction with diabetes treatments, and it has no existing psychometric examination on its factor structure. (WE-CARE measures concepts such as communication, medical care, emotional distress, and role function differently from the Pediatric Inventory for Parents.) The Insulin Pump Therapy Satisfaction Questionnaire measures parents' satisfaction with their child's implantable insulin pump [11]. Although disease-specific, this questionnaire was only tested for internal consistency reliability (α = 0.69, which is less than the acceptable level of 0.70), and its scope is limited to the insulin pump.

Given the lack of a well-validated instrument to assess the well-being and treatment satisfaction for parents of children with type 1 diabetes, we developed and validated a new measure: the **WE**ll-being and Satisfaction of **CARE**givers of Children with Diabetes Questionnaire. WE-CARE measures the psychosocial well-being and treatment satisfaction of parents who have a child with type 1 diabetes. Specific topics covered in WE-CARE include overall burden of the parents/caregivers, their anxiety and stress, influence on their social life, work, and families, and satisfaction with diabetes treatment (such as administering and preparation of injections, carrying, storing and disposing of the insulin, and flexibility in the use of insulin).

## Methods

The development of WE-CARE began with a literature review, followed by one-on-one interviews conducted in New York, NY, and Philadelphia, PA, with four pediatricians, 20 children, and their primary caregivers. Findings from the interviews were reviewed by a panel of four pediatricians (distinct from the ones interviewed) and one child psychologist, after which a draft questionnaire was developed. Initial assessments of item and content validity resulted in a self-administered questionnaire consisting of 68 items, which took about 20 minutes to complete. A validation study comparing results on WE-CARE to results on two previously validated quality-of-life (but non-diabetes-specific) questionnaires was then conducted.

### Study subjects

Subjects were recruited into the validation study from diabetes clinics, specialists, and clinical investigators located in four US cities: Buffalo, NY; Minneapolis, MN; Oklahoma City, OK; and Tallahassee, FL. The study protocol was approved by the institutional review board at each center. Study participants (the children and their parents/caregivers) were identified by clinical investigators and given a detailed explanation of the protocol and corporate funding of the study. Subjects provided informed written consent, and child subjects were required to have written permission and informed consent from a parent or legal guardian.

Parents or caregivers of children with type 1 diabetes aged 6 to 11 years were eligible for entry into the study. Parents received compensation of $100, and children were given an age-appropriate gift valued at $50. Only one parent or caregiver per child participated; the participant was required to be the primary caregiver of the child, and had to be willing to participate throughout the course of the study and to complete the questionnaires at Weeks 0 and 2.

The questionnaires were administered at the diabetes clinic, except for one site (Buffalo), where they were administered at the parents' home. The children were required to have had a diagnosis of type 1 diabetes for at least one year, and have been taking a stable regimen of at least two subcutaneous injections of insulin or insulin analog (16–150 IU per day) for two months prior to study entry. They must have had a glycosylated hemoglobin (HbA_1c_) measurement within the past two months.

### Data collection

Clinicians reported child medical history, including year of type 1 diabetes diagnosis, HbA_1c _values, height, weight, history of diabetes complications, insulin treatment regimens, other medications, and other medical conditions. Parents/caregivers and children were excluded from the study if they had any clinically significant major organ system disease or psychiatric condition, or had experienced major life stress or health changes in the two weeks between baseline (test) and Week 2 (retest).

The study was conducted between January and August 2002. Parents/caregivers were asked to complete WE-CARE at baseline (Week 0) and after two weeks (Week 2), together with the 36-item Short Form of the Medical Outcomes Study (SF-36) [12,13] and the 50-item Child Health Questionnaire-Parent Form (CHQ-PF50) [14,15]. The SF-36 measures eight dimensions, including physical functioning, role limitations-physical, pain, general health perception, role limitations-emotional, vitality, social functioning, and mental health. It can be summarized into two component scores: physical component summary and mental component summary.

The SF-36 has been tested and validated in a wide range of patient groups [12,13]. The CHQ-PF50 is a global health-related quality-of-life instrument for parents of children aged 5 to 18 years [14,15]. It is intended to measure parent's understanding of his or her child's general quality of life based on 8 constructs, including physical functioning, role limitations-physical, pain, general health perceptions, role limitations-emotional, behavior, mental health, and self-esteem.

An additional four constructs assess the parents' perspectives of how the child's health affects them and their family through impact on parent's time, impact on parent's emotions, impact on family, and family cohesion. All analyses of the WE-CARE validation were based on assessments at Week 0, except for test-retest reliability, which incorporated assessments at Week 0 and Week 2.

### Factor analysis

Initial factor analyses for item reduction were conducted with the aim of establishing which of the provisional 68 items in WE-CARE belonged to domains or conceptual areas and which items should be retained. Items were deleted if they loaded on two or more factors, had a correlation coefficient of < 0.40 with their own factor, or had a high (> 70% of response) floor-ceiling effect – unless the item was considered clinically relevant. Response options to each item (question) were based on a five-point ordinal scale, with higher response codes being more favorable. All domain scores, once established, were transformed onto a 0 to100 scale, with 100 being most favorable.

### Reliability and validity

WE-CARE was assessed for the following battery of psychometric properties: floor and ceiling effects (percentage of subjects scoring the lowest and highest scores possible), internal consistency reliability (satisfied if Cronbach's α coefficient ≥ 0.70), test-retest reliability (satisfied if intra-class correlation coefficient ≥ 0.70), item-convergent validity (satisfied if item-scale correlation achieved ≥ 0.40), item-discriminant validity (items correlated more highly with their own scale than with any other scale), scale-scale correlations (domains are related but distinct), concurrent validity, divergent validity, known-groups validity, and clinical validity.

Concurrent validity was examined through an analysis of correlation between WE-CARE scores and mental component summary of the SF-36. We expected that parents who scored higher on WE-CARE (better psychosocial well being and higher satisfaction with their child's diabetes treatment) would have higher scores on the mental component summary of the SF-36. This is because psychosocial well-being is closely related to mental health, and diabetic control in children has been linked to parental depression and family instability [4]. We also expected to see moderate to high correlations (> 0.4) between the WE-CARE scores and the four CHQ-PF50 scales that assessed the impact of the child's health on the parents and family,

Divergent validity was assessed by correlating the WE-CARE scores with the physical component summary of the SF-36 and by comparing the WE-CARE scores by child age and gender. Child age was dichotomized (6–8 years and 9–11 years), and then WE-CARE scores were compared between the 2 age groups with a *t *test. Because the WE-CARE primarily focuses on parents' psychosocial well-being and the physical component summary of the SF-36 assessed parents' physical health, we did not expect that these two scores would be highly correlated. Neither did we expect that the WE-CARE scores would be different among children with different ages and genders, as we are not aware of any published data or empirical evidence that support this.

Known-groups validity was analyzed through the comparison of the WE-CARE scores across groups of children with type 1 diabetes with different health status. Because parents have reported higher levels of stress when their child had poor health, i.e., chronic disease, and children's health can directly impact parents' perception about their child's treatment, we expected that parents of healthier children with type 1 diabetes would report higher WE-CARE scores. The health status of the child's type 1 diabetes was assessed using an item of the CHQ-PF50 ("In general, would you say your child's health is....?") We grouped responses into excellent/very good, good, and fair/poor and compared WE-CARE scores across these three groups.

Clinical validity was investigated by correlating child HbA_1c _level with WE-CARE scores. Based on the previous findings that diabetic control in children was linked to parental depression and parental life satisfaction [4,16], we expected that higher HbA_1c _scores in children (poor diabetic control) would be associated with lower WE-CARE score.

SAS/STAT^® ^(SAS Institute, Cary, NC) software was used for the assessment of factor analysis and for clinical and known-groups validity. Multitrait Analysis Program-Revised software [17] was used for the assessment of other psychometric elements. For all tests, a significance level of 0.05 was used.

## Results

One hundred sixteen parents and their children were included in the study. Approximately 90% of the parents/caregivers were female, usually mothers, with a mean age of 37.1 years (Table 1). The majority of the subjects were white. Because only one of the adults taking part in the study was not the parent of the participating child, "parents/caregivers" will hereafter be referred to simply as "parents."

**Table 1:** Subject characteristics

	**Parent (N = 116)**	**Child (N = 116)**
Age (years), mean ± SD	37.1 ± 6.3	8.6 ± 1.7
Gender (male %)	9.5	52.6
Relation to child (%):		
Mother	90.5	NA
Father	8.6	NA
Grandparent	0.9	NA
Ethnicity (%):		
White	86.2	86.2
Other	13.8	13.8
Work status (%):		
Not working due to child's health	4.3	NA
Not working for other reasons	6.0	NA
Looking for work	2.6	NA
Working full- or part-time	75.9	NA
Full-time homemaker	11.2	NA
Income in United States dollars (%):		
<$30,000	29.3	NA
$30,000–$59,000	31.0	NA
≥$60,000	37.9	NA
Body mass index (kg/m^2^), mean ± SD	NA	19.2 ± 4.1
HbA1c (%), mean ± SD	NA	8.4 ± 1.3
Duration of diabetes (%):		
1–3 years	NA	55.2
4–10 years	NA	44.8
Insulin regimen, n (%)		
Lispro 2 times daily/NPH 2 times daily	NA	45 (38.8%)
Lispro 3 times daily/NPH 2 times daily	NA	17 (14.7%)
Lispro 3 times daily/Glargine 1-time daily	NA	10 (8.6%)
Lispro 3 times daily	NA	8 (6.9%)
Other	NA	36 (31.0%)
Children with other medical conditions (%)*	NA	37

NA, not applicable; HbA1c, Hemoglobin A1c; NPH, neutral protamine Hagedorn.

*Other medical conditions were clinician-reported and included conditions such as allergies, asthma, attention deficit hyperactivity disorder, bronchitis, ear problems, heart murmur, and thyroid problems.

Parents were required to have completed all items in each scale to be included in the factor analysis. There were no missing items in 81.03% (n = 94) of questionnaires at Week 0 and in 90.27% (n = 102) at Week 2. The mean percentage of missing items per parent was 0.87% (range, 0%–68%) at Week 0 and 0.29% (range, 0%–15%) at Week 2.

Preliminary factor analyses for item reduction were examined, and 31 of the initial 68 items were deleted for loading relatively high on all the factors (≥ 0.40) or relatively low on all the factors (< 0.40). Items that had low variability in response (floor or ceiling effect) or that were worded ambiguously were also deleted. The remaining 37 items were grouped into concepts (factors) using exploratory factor analysis (with Promax rotation). Eigenvalues for the first four factors were 12.08, 3.21, 2.18, and 1.78, respectively. After that, beginning with the fifth factor with an eigenvalue of 1.21, the scree plot showed a break that suggested a four-factor solution. The first four factors explained about 73% of the common variance in the data. Among the several types of factor structures that were fit and evaluated, the four-factor solution gave the best results based on its standardized pattern coefficients.

Results were grouped into four multi-item scales: Psychosocial Well-being (13 items), Ease of Insulin Use (9 items), Treatment Satisfaction (9 items), and Acceptance of Insulin Administration (6 items). The labeling of these scales was based on the consensus of the research team, including the 4 authors who are external to Pfizer. These scores were combined (with equal weighting) to give a domain and a total score. The factor loadings for the final factor analysis are provided in Table 2. The Appendix contains the WE-CARE questionnaire with its 37-items categorized by their domain and the response category for each item [see Additional file 1].

**Table 2:** WE-CARE factor analysis, standardized regression coefficients, Promax rotation, number of factors fixed at 4

Item	Factor 1: Psychosocial Well-being	Factor 2: Ease of Insulin Use	Factor 3: Treatment Satisfaction	Factor 4: Acceptance of Insulin Administration
The burden of care is overwhelming	**0.47**	0.01	0.06	0.24
I get frustrated a lot	**0.58**	-0.00	0.05	0.11
I feel depressed	**0.67**	-0.15	0.02	0.14
Was a burden on my marriage	**0.82**	-0.01	-0.21	0.11
Made me spend less time with my other children or other family members	**0.66**	0.25	-0.08	-0.03
Made me spend less time at work	**0.59**	0.11	0.02	-0.25
Interrupted my work	**0.65**	0.12	0.04	-0.12
Interrupted my social activities	**0.67**	0.14	-0.03	-0.093
Your work (job) situation	**0.68**	0.04	0.02	-0.04
Your leisure time activities	**0.67**	0.00	0.17	0.03
Your marriage/partnership	**0.81**	-0.16	-0.07	0.17
Your relationship with your children	**0.49**	-0.01	0.18	0.08
Your sexual life	**0.75**	-0.17	0.03	0.03
Disposing of used supplies	0.05	**0.62**	-0.07	0.24
Carrying insulin and supplies	0.05	**0.70**	-0.12	0.31
Storing insulin	0.03	**0.50**	-0.14	0.36
Easy/difficult to prepare the insulin dose	-0.09	**0.52**	0.17	-0.03
Easy/difficult to use the insulin	-0.02	**0.58**	0.24	-0.05
Easy/difficult to carry insulin	-0.06	**0.84**	0.12	-0.06
Easy/difficult to carry supplies	-0.05	**0.83**	0.08	-0.03
I prefer to stay home rather than use insulin away from home	0.15	**0.59**	-0.09	0.21
I find it difficult to administer the insulin away from home	0.01	**0.70**	-0.00	0.19
I worry about complications of diabetes	0.12	-0.08	**0.40**	0.21
Flexibility in your daily activities	0.30	0.26	**0.53**	-0.15
Flexibility in planning your social activities	0.30	0.28	**0.51**	-0.07
Flexibility around mealtimes	0.18	0.18	**0.56**	-0.05
Overall, satisfaction with the insulin treatment	0.01	0.20	**0.60**	0.01
I find the time it takes for each dosing acceptable	0.03	0.09	**0.39**	0.20
I would recommend the current insulin regimen to others	-0.09	-0.10	**0.80**	0.13
I want my child to continue using the current insulin regimen	-0.14	0.01	**0.79**	0.11
My child is compliant with the current insulin regimen	-0.06	-0.05	**0.47**	0.24
The pain that giving insulin causes your child	0.05	-0.03	0.33	**0.51**
Preparing insulin for administration	-0.17	0.25	0.16	**0.54**
Having to administer insulin to your child	0.03	-0.17	0.37	**0.67**
Administering insulin prior to meals	-0.00	0.33	0.03	**0.63**
Administering insulin in public places	-0.01	0.37	-0.04	**0.60**
Administering insulin at home	0.14	0.00	0.03	**0.71**

A boldface number represents the largest coefficient of that item on a given factor.

All domains exceeded the minimum standard of 0.70 for internal consistency (range: 0.84–0.95) and test-retest reliability (range: 0.80–0.88). No significant floor or ceiling effects were observed, and moderate scale-scale correlations (range: 0.44–0.61) (Table 3) showed the domains to be related but distinct.

**Table 3:** WE-CARE Inter-scale correlations

**Subscale correlation with subscale**	**Correlation**
Psychosocial Well-being with Ease of Insulin Use	0.54
Psychosocial Well-being with Treatment Satisfaction	0.50
Psychosocial Well-being with Acceptance of Insulin Administration	0.44
Ease of Insulin Use with Treatment Satisfaction	0.56
Ease of Insulin Use with Acceptance of Insulin Administration	0.61
Treatment Satisfaction with Acceptance of Insulin Administration	0.55

The validity of WE-CARE was assessed by a number of psychometric tests. In item-convergent validity analysis, 97% of the items achieved the standard of item-scale correlations of ≥ 0.40 (range: 0.35–0.78). All WE-CARE items were significantly (*P *< 0.05) more highly correlated with the total score from their own domain (after removing the item from that domain) than with the total score from any other domain, thus satisfying the test of item-discriminant validity. For concurrent validity test, all WE-CARE domain scores were significantly and meaningfully correlated with the SF-36 mental component summary score and with the CHQ-PF50 impact on parent's time, impact on parent's emotions, and family impact. All WE-CARE domain scores except acceptance of insulin administration were significantly correlated with the family cohesion construct (Table 4). In contrast, the correlations between each domain of WE-CARE and the SF-36 physical component summary were much lower and not statistically significant (Table 4), and no significant differences in WE-CARE scores were found by child age (*P *> 0.05) or gender (*P *> 0.05). These findings supported divergent validity.

**Table 4:** Pearson correlations (*P *values) between WE-CARE domain score and SF-36 Summary Scale and CHQ-PF 50 Parent and Family Impact scales (N = 115)

**WE-CARE Domain**	**SF-36**		**CHQ-PF50**			
	
	Standardized Physical Component Scale	Standardized Mental Component Scale	Impact on Parent's Time	Impact on Parent's Emotions	Family Impact	Family Cohesion
Psychosocial Well-being	0.12 (0.21)	0.65 (< 0.0001)	0.60 (< 0.0001)	0.54 (< 0.0001)	0.68 (< 0.0001)	0.28 (0.0025)
Ease of Insulin Use	0.12 (0.22)	0.31 (0.0008)	0.44 (< 0.0001)	0.35 (0.0002)	0.43 (< 0.0001)	0.28 (0.0027)
Treatment Satisfaction	0.10 (0.30)	0.35 (0.0002)	0.41 (< 0.0001)	0.51 (< 0.0001)	0.41 (< 0.0001)	0.30 (0.0011)
Acceptance of Insulin Administration	0.04 (0.65)	0.28 (0.0029)	0.41 (< 0.0001)	0.30 (0.0012)	0.42 (< 0.0001)	0.13 (0.2126)
WE-CARE Total Score	0.12 (0.19)	0.55 (< 0.0001)	0.61 (< 0.0001)	0.55 (< 0.0001)	0.64 (< 0.0001)	0.32 (0.0006)

**Figure 1:**
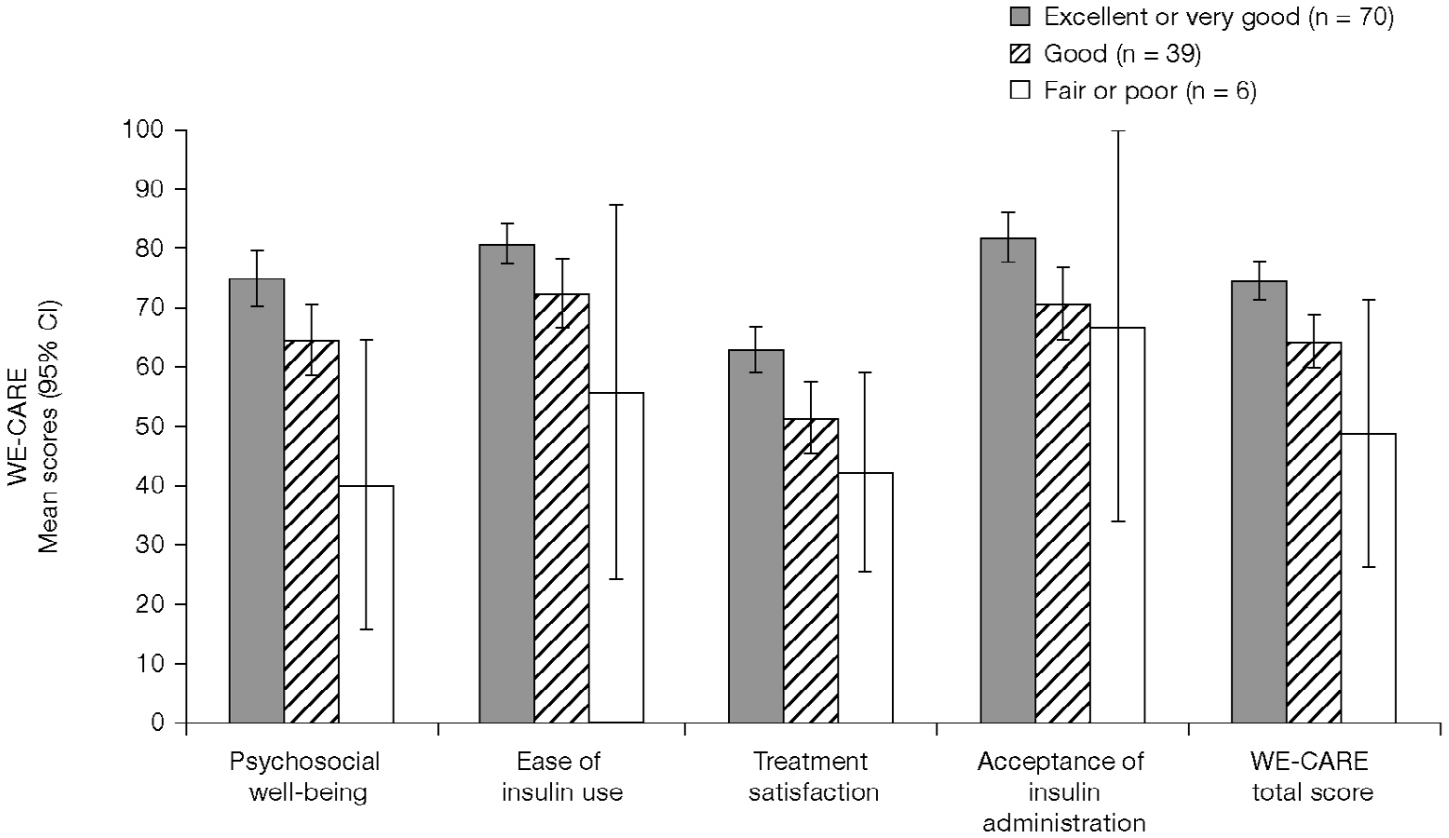
WE-CARE scores according to parent's rating of child's health* (scales range from 0 to 100, with higher scores being more favorable). *P < 0.01 in testing the difference in means among the three health groups (item 1.1 of the CHQ-PF50) for all scales and Total Score of WE-CARE. WE-CARE, WEll-being and Satisfaction of CAREgivers of Children with Diabetes Questionnaire; CI, confidence interval; CHQ-PF50, Child Health Questionnaire-Parent Form.

As for known group validity test, the WE-CARE domain and summary scores were lower when parents rated their child's general health to be worse and the score differences across the three health groups were statistically significant (*P *< 0.01) (Figure 1). Finally, in assessing clinical validity, as child HbA_1c _levels increased, WE-CARE scores decreased. Statistically significant negative correlations were observed for Psychosocial Well-being (r = -0.26; *P *< 0.01), Treatment Satisfaction (r = -0.20; *P *= 0.03), Acceptance of Insulin Administration (r = -0.21; *P *= 0.03), and WE-CARE Total Score (r = -0.27; *P *< 0.01), with a trend toward significance for Ease of Insulin Use (r = -0.18; *P *= 0.06).

## Discussion

Few, if any, studies have assessed quantitatively the well-being or treatment experiences with diabetes regimens in parents with a child with type 1 diabetes. Qualitatively, in our semi-structured interviews, parents often indicated that their child's physician or health care team never discussed their well-being or satisfaction with them. The parent's perceptions about the child's insulin regimen could have a significant impact on both the child's and the parent's well-being and, ultimately, whether a certain regimen will be successful.

Evidence indicates that following item reduction by factor analysis, WE-CARE is both reliable and valid among parents of children with type 1 diabetes. The four multi-item domains of WE-CARE were found to be psychometrically robust – they had negligible floor and ceiling effects, excellent internal consistency and test-retest reliability, high item-discriminant validity, and good concurrent, divergent, known-group and clinical validity; moderate interscale correlations among the four WE-CARE domains indicate that the concepts they measure are related but distinct.

The factor analysis reveals four distinct factors of the WE-CARE. One encompasses an array of psychosocial elements, and the other three cover various aspects of insulin treatment. While related, these three factors measure different aspects of insulin treatment. The distinct factor structure of WE-CARE has been supported by the item-discriminant validity tests in which constituent items were more correlated with their own domains than with any other domains.

Regarding the divergent validity analyses, WE-CARE scores were not different among children with different ages and genders. This is expected as parents' well-being and satisfaction with their child's treatment should not be influenced by their child's age and gender. The lack of differences in WE-CARE scores by child age and gender suggests that the questionnaire is not expected to produce biased scores on gender and the 6- to 11-year-old age range.

WE-CARE was able to discriminate between parents' reports of their child's health. Differences in WE-CARE scores across the different health groups were anticipated *a priori*, as child health was expected to be linked to parents' psychosocial well-being and to influence parents' perceptions about their child's treatment. WE-CARE scores were also found to correlate with the mental component summary of the SF-36 and with the impact of the child's health on parent's time and emotions and on the family, as measured by the CHQ-PF50. Because both the psychosocial domain of WE-CARE and the mental component summary of the SF-36 measure similar concepts, a sizeable correlation between the two scores was expected. Similarly, we were expecting, and found, moderate-to-high correlations between WE-CARE satisfaction domains and the impact on parents and family. Also within our expectation is the correlation between the WE-CARE scores and child's HbA_1c _level, as glycemic control in child has been linked to parental depression and parental life satisfaction [4,16].

We acknowledge that validation of any instrument is an ongoing process and our validation of WE-CARE is an essential first step toward a fuller validation of this instrument. Although promising, our preliminary validation in this report deserves qualifications in three areas: (1) although we correlated WE-CARE with the mental component summary of SF-36 and the CHQ-PF50 impact scales in our concurrent validity tests, we did not correlate WE-CARE with other relevant measures such as the Insulin Pump Therapy Satisfaction Questionnaire and the Pediatric Inventory for Parents; (2) the questionnaire was designed broadly enough to apply to parents of children treated with insulin pump therapy or inhaled insulin regimens, but the current study includes mainly parents of children treated with subcutaneous insulin regimens; and (3) no record was obtained on the individuals who were screened and eligible but chose not to participate; therefore, the extent of possible selection or response bias cannot be assessed.

## Conclusion

Based on this initial psychometric validation of WE-CARE, use of the 37-item questionnaire (four multi-item domains) in conjunction with continued research is recommended. Concerted efforts are encouraged in several areas such as the evaluation of the responsiveness and sensitivity of WE-CARE to changes over time, and in the incorporation of WE-CARE into routine clinical assessments and trials to improve understanding and interpretation of individual parent scores. Although the results of this study appear to be robust, the relatively limited sample size warrants confirmation of the factor structure by a study with a larger group (≥ 185 subjects = 37 items × 5 subjects/item). In addition, further testing with parents of children under age 6 and over age 11 is recommended if WE-CARE will be used in those populations.

Nonetheless, results of this study suggest that WE-CARE provides a reliable and valid measure of parents' treatment-related psychosocial well-being and satisfaction in relation to their child's diabetes. The questionnaire could, therefore, be a useful tool to initiate and monitor feedback systematically among parents, children, physicians, nurses, and other health care professionals in clinical practice and, in addition, to assess and explain changes over time within and between different insulin regimens in clinical practice or research. Use of WE-CARE may enhance understanding of parental quality of life and their children's treatment, contribute to improved treatment strategies that maximize compliance and satisfaction, and, ultimately, improve the well-being of children with diabetes.

## Abbreviations

CHQ-PF50, Child Health Questionnaire-Parent Form (50 item)

HbA1c, glycosylated hemoglobin

NPH, neutral protamine Hagedorn

PDQOL, Parents Diabetes Quality of Life Questionnaire

SF36, Short Form of the Medical Outcomes Study (36 items)

WE-CARE, WEll-being and Satisfaction of CAREgivers of Children with Diabetes Questionnaire

## Competing interests

Joseph C. Cappelleri, Robert A. Gerber, and Xuemei Luo are employees of Pfizer Inc. Teresa Quattrin has received consultant fees from Pfizer. Rosemarie Deutschmann was contracted by Pfizer to assist with the design and coordination of this study. Rob Arbuckle and Linda Abetz are employees of MapiValues Ltd, which was contracted by Pfizer to conduct and analyze this research.

## Authors' contributions

All authors made intellectual contributions and contributed to the writing of the manuscript.

JC and RG conceived of the study instrument, participated in analyzing study results, and helped to draft the manuscript. TQ participated in design and coordination of the study and helped to draft the manuscript. RD participated in the design and coordination of the study. XL contributed statistical analysis and helped to draft the manuscript. RA and LA participated in the design and coordination of the study, analyzed and reported its results.

## Supplementary Material

Additional file 1 Appendix: The WEll-being and Satisfaction of CAREgivers of Children with Diabetes Questionnaire. The parent questionnaire upon which the study was based. Click here for file
