# Supplementary material for: Development and validation of the WEll-being and Satisfaction of CAREgivers of Children with Diabetes Questionnaire (WE-CARE)
Source: Health Qual Life Outcomes. 2008 Jan 18;6:3. doi: 10.1186/1477-7525-6-3 (PMC2262877; doi:10.1186/1477-7525-6-3)
Supplement: Additional file 1 — Appendix: The WEll-being and Satisfaction of CAREgivers of Children with Diabetes Questionnaire. The parent questionnaire upon which the study was based. [file 1477-7525-6-3-S1.doc]

**Appendix**

The **WE**ll-being and Satisfaction of **CARE**givers of Children with Diabetes Questionnaire

This questionnaire is about you and the care that you provide for your child with diabetes. It is very important for us to understand your experiences managing your child's diabetes.

Please read the following questions carefully and answer by making an X at the place that corresponds to your opinion -- there are no right or wrong answers. Thank you for your help!

**Think about the care that you provide for your child’s diabetes over the past 4 weeks. Please place an X in the box that indicates how much you agree or disagree with each statement.**

| Over the past 4 weeks… | Strongly Agree | Agree | Neither Agree nor Disagree | Disagree | Strongly Disagree |
| --- | --- | --- | --- | --- | --- |
| 1. The burden of care is overwhelming* | 🞏 | 🞏 | 🞏 | 🞏 | 🞏 |
|  |  |  |  |  |  |
| 2. I get frustrated a lot* | 🞏 | 🞏 | 🞏 | 🞏 | 🞏 |
|  |  |  |  |  |  |
| 3. I worry about complications of diabetes^†^ | 🞏 | 🞏 | 🞏 | 🞏 | 🞏 |
|  |  |  |  |  |  |
| 4. I feel depressed* | 🞏 | 🞏 | 🞏 | 🞏 | 🞏 |
|  |  |  |  |  |  |

**Think about the current insulin regimen that your child is using.** For each question, place an X in the box that corresponds to the level of bother or burden to you.

| **How much of a burden or bother is the following:** | Almost always/  Always a burden | Most of the time a burden | Sometimes a burden | Occasionally a burden | Almost never/  Never a burden |
| --- | --- | --- | --- | --- | --- |
| 5. The pain that giving insulin causes your child^‡^ | 🞏 | 🞏 | 🞏 | 🞏 | 🞏 |
|  |  |  |  |  |  |
| 6. Preparing insulin for administration^‡^ | 🞏 | 🞏 | 🞏 | 🞏 | 🞏 |
|  |  |  |  |  |  |
| 7. Having to administer insulin to your child^‡^ | 🞏 | 🞏 | 🞏 | 🞏 | 🞏 |
|  |  |  |  |  |  |
| 8. Administering insulin prior to meals^‡^ | 🞏 | 🞏 | 🞏 | 🞏 | 🞏 |
|  |  |  |  |  |  |
| 9. Administering insulin in public places^‡^ | 🞏 | 🞏 | 🞏 | 🞏 | 🞏 |
|  |  |  |  |  |  |
| 10. Administering insulin at home^‡^ | 🞏 | 🞏 | 🞏 | 🞏 | 🞏 |
|  |  |  |  |  |  |
| 11. Disposing of used supplies^§^ | 🞏 | 🞏 | 🞏 | 🞏 | 🞏 |
|  |  |  |  |  |  |
| 12. Carrying insulin and supplies^§^ | 🞏 | 🞏 | 🞏 | 🞏 | 🞏 |
|  |  |  |  |  |  |
| 13. Storing insulin^§^ | 🞏 | 🞏 | 🞏 | 🞏 | 🞏 |
|  |  |  |  |  |  |

**Think about the care that you provide for your child’s diabetes over the past 4 weeks and the impact that it has on you socially. Place an X in the box that indicates how much you agree or disagree with each statement.**

| Over the past 4 weeks, the care for my child with diabetes. . . | Strongly Agree | Agree | Neither Agree nor Disagree | Disagree | Strongly Disagree |
| --- | --- | --- | --- | --- | --- |
| 14. Was a burden on my marriage* | 🞏 | 🞏 | 🞏 | 🞏 | 🞏 |
|  |  |  |  |  |  |
| 15. Made me spend less time with my other children or other family members* | 🞏 | 🞏 | 🞏 | 🞏 | 🞏 |
|  |  |  |  |  |  |
| 16. Made me spend less time at work* | 🞏 | 🞏 | 🞏 | 🞏 | 🞏 |
|  |  |  |  |  |  |
| 17. Interrupted my work* | 🞏 | 🞏 | 🞏 | 🞏 | 🞏 |
|  |  |  |  |  |  |
| 18. Interrupted my social activities* | 🞏 | 🞏 | 🞏 | 🞏 | 🞏 |

**Place an X in the box that indicates how much or how little of an impact being a parent that takes care of a child with diabetes has been over the past 4 weeks.**

| **Over the past four weeks, how much of an impact has taking care of your child with diabetes had on…** | Very Much | Much | Neither much nor little | Little | Very Little |
| --- | --- | --- | --- | --- | --- |
| 19. Your work (job) situation* | 🞏 | 🞏 | 🞏 | 🞏 | 🞏 |
|  |  |  |  |  |  |
| 20. Your leisure time activities* | 🞏 | 🞏 | 🞏 | 🞏 | 🞏 |
|  |  |  |  |  |  |
| 21. Your marriage/partnership* | 🞏 | 🞏 | 🞏 | 🞏 | 🞏 |
|  |  |  |  |  |  |
| 22. Your relationship with your children* | 🞏 | 🞏 | 🞏 | 🞏 | 🞏 |
|  |  |  |  |  |  |
| 23. Your sexual life* | 🞏 | 🞏 | 🞏 | 🞏 | 🞏 |

**Place an X in the box that best indicates your opinion.**

| 24. How easy or difficult is it for you to prepare the insulin dose? (R)^§^ | | | | |
| --- | --- | --- | --- | --- |
| Very Easy | Easy | Neither Easy nor Difficult | Difficult | Very Difficult |
| 🞏 | 🞏 | 🞏 | 🞏 | 🞏 |
|  |  |  |  |  |
| 25. How easy or difficult is the insulin to use? (R)^§^ | | | | |
| Very Easy | Easy | Neither Easy nor Difficult | Difficult | Very Difficult |
| 🞏 | 🞏 | 🞏 | 🞏 | 🞏 |
|  |  |  |  |  |
| 26. How easy or difficult is it for you to carry insulin? (R)^§^ | | | | |
| Very Easy | Easy | Neither Easy nor Difficult | Difficult | Very Difficult |
| 🞏 | 🞏 | 🞏 | 🞏 | 🞏 |
|  |  |  |  |  |
| 27. How easy or difficult is it for you to carry supplies? (R)^§^ | | | | |
| Very Easy | Easy | Neither Easy nor Difficult | Difficult | Very Difficult |
| 🞏 | 🞏 | 🞏 | 🞏 | 🞏 |
|  |  |  |  |  |
| 28. How much flexibility in your daily activities does the insulin regimen give you? (R)^†^ | | | | |
| Very Flexible | Flexible | Neither Flexible nor Inflexible | Inflexible | Very Inflexible |
| 🞏 | 🞏 | 🞏 | 🞏 | 🞏 |
|  |  |  |  |  |
| 29. How much flexibility in planning your social activities does the insulin regimen give you? (R)^†^ | | | | |
| Very Flexible | Flexible | Neither Flexible nor Inflexible | Inflexible | Very Inflexible |
| 🞏 | 🞏 | 🞏 | 🞏 | 🞏 |
|  |  |  |  |  |
| 30. How much flexibility around mealtimes does the insulin regimen give you? (R)^†^ | | | | |
| Very Flexible | Flexible | Neither Flexible nor Inflexible | Inflexible | Very Inflexible |
| 🞏 | 🞏 | 🞏 | 🞏 | 🞏 |
|  |  |  |  |  |
| 31. Overall, how satisfied are you with the insulin treatment being used? (R)^†^ | | | | |
| Very Satisfied | Satisfied | Neither satisfied nor dissatisfied | Dissatisfied | Very Dissatisfied |
| 🞏 | 🞏 | 🞏 | 🞏 | 🞏 |

**Please place an X in the box that indicates how much you agree or disagree with each question.**

|  | Strongly Agree | Agree | Neither Agree nor Disagree | Disagree | Strongly Disagree |
| --- | --- | --- | --- | --- | --- |
| 32. I find the time it takes for each dosing acceptable (R)^†^ | 🞏 | 🞏 | 🞏 | 🞏 | 🞏 |
|  |  |  |  |  |  |
| 33. I prefer to stay home rather than use insulin away  from home^§^ | 🞏 | 🞏 | 🞏 | 🞏 | 🞏 |
|  |  |  |  |  |  |
| 34. I find it difficult to administer the insulin away from home^§^ | 🞏 | 🞏 | 🞏 | 🞏 | 🞏 |
|  |  |  |  |  |  |
| 35. I would recommend the current insulin regimen  to others (R)^†^ | 🞏 | 🞏 | 🞏 | 🞏 | 🞏 |
|  |  |  |  |  |  |
| 36. I want my child to continue using the current insulin regimen (R)^†^ | 🞏 | 🞏 | 🞏 | 🞏 | 🞏 |
|  |  |  |  |  |  |
| 37. My child is compliant with the current insulin (R)^†^ | 🞏 | 🞏 | 🞏 | 🞏 | 🞏 |

(WE-CARE,© 2000 Pfizer Inc. For use with permission of Pfizer.)

*****Psychosocial well-being (items 1, 2, 4, 14, 15, 16, 17, 18, 19, 20, 21, 22, 23)

^†^Treatment satisfaction (items 3, 28, 29, 30, 31, 32, 35, 36, 37)

^‡^Acceptance of insulin administration (items 5, 6, 7, 8, 9, 10)

^§^Ease of insulin use (items 11, 12, 13, 24, 25, 26, 27, 33, 34)

(R) indicates that the item score needs to be reversed such that a higher score means more satisfaction.
